# A method for rapid 3D scanning and replication of large paleontological specimens

**DOI:** 10.1371/journal.pone.0179264

**Published:** 2017-07-05

**Authors:** Anshuman J. Das, Denise C. Murmann, Kenneth Cohrn, Ramesh Raskar

**Affiliations:** 1 MIT Media Lab, Massachusetts Institute of Technology, Cambridge, MA, United States of America; 2 Murmann Dental Health, Naperville, IL, United States of America; 3 KFCForensics, 422 Teague Trail, Lady Lake, FL, United States of America; Seconda Universita degli Studi di Napoli, ITALY

## Abstract

We demonstrate a fast and cost-effective technique to perform three dimensional (3D) scanning and replication of large paleontological specimens, in this case the entire skull of a *Tyrannosaurus rex* (T.rex) with a volume in the range of 2 m^3^. The technique involves time-of-flight (TOF) depth sensing using the Kinect scanning module commonly used in gesture recognition in gaming. Raw data from the Kinect sensor was captured using open source software and the reconstruction was done rapidly making this a viable method that can be adopted by museums and researchers in paleontology. The current method has the advantage of being low-cost as compared to industrial scanners and photogrammetric methods but also of accurately scanning a substantial volume range which is well suited for large specimens. The depth resolution from the Kinect sensor was measured to be around 0.6 mm which is ideal for scanning large specimens with reasonable structural detail. We demonstrate the efficacy of this method on the skull of FMNH PR 2081, also known as SUE, a near complete T.rex at the Field Museum of Natural History.

## Introduction

The field of paleontology has transformed in the last few years as a result of the developments in 3D scanning technology and rendering software that have enhanced the quality of virtual models [1–4]. Conventionally, a photograph is utilized for research purposes which has its benefits but also has limited application. A two dimensional (2D) image is easy to capture, interpret and is still a useful method of analysis in paleontology research [5–7]. However, a 2D image cannot capture the details regarding depth of the scene. Recent studies have shown that 3D scanning and analysis of specimens can provide rich information which can be beneficial in a range of studies [8]. These techniques are increasingly seen in museums and research labs due to the compact nature of some of the imaging devices [3, 4]. 3D scanning can provide depth maps in a non-invasive, non-contact manner which is attractive for studying paleontological specimens due to their delicate physical properties. For instance, it has been used to estimate the mass of dinosaurs by combining it with computer modeling [9]. It has also been used to create virtual skeletons for different fauna for comparative purposes [10]. Other examples of 3D scanning in related fields include typology [11], pottery studies [12] and footprint analysis in archaeology [13].

At the heart of 3D imaging technology is the 3D scanner itself. There are several approaches to perform 3D scanning from structured light scanners to computed tomography (CT). However, most of these scanners are industrial or clinical grade instruments and are generally very expensive and bulky. Structured light scanners need calibration and are inherently expensive due to the requirement of a laser projector and a high end camera to capture the images. There are reports of using structured light based 3D scanning for fossils of the size of several tens of centimeters [14] but not large specimens like T.rex skulls [10]. Several other reports have demonstrated the use of CT imaging due to its ability to study internal details of specimens. However, CT scanners are expensive and the imaging is done at a clinical facility [15, 16]. Additionally, most studies have used these techniques on small specimens due to the complexity of the scanner and also restriction of the data size that can be handled by the software for large specimens. For instance, a high resolution dental scanner would not be able to handle the large data size when scanning the jaw of a T.rex. Hence, there are limitations in the volume of the object that can be scanned with these methods, the ease of setup and processing the data. Furthermore, the software for these industrial scanners is proprietary making it inaccessible to researchers and museums. Although there have been some reports on the use of free open source photogrammetric software for 3D imaging, the process is cumbersome requiring a large amount of data to reconstruct the models [17]. Hence there is a need for a technique that is accurate, low-cost, easy to implement, has open source software capability and can be adapted for large scale paleontological scanning.

We propose a new technique that provides high quality 3D reconstructions of large specimens with relative ease. We used the Kinect v2 TOF sensor to perform 3D scanning of large paleontological specimens for the first time. Kinect has traditionally been used in gesture recognition [18–20] in gaming, computer graphics [21] and more recently in 3D scanning [22–24]. There has been one earlier report that used Kinect v1 for paleontological specimens but the reconstructions were noisy and smoothing the data resulted in loss of features [14]. Kinect v1 uses structured light imaging in contrast to Kinect v2 that is based on TOF imaging which has significantly improved since the report by Falkingham [14]. The sensor technology in Kinect v2 is not only superior to Kinect v1 but also the computation aspect has improved providing real-time high quality reconstructions. The Kinect has shown to be a promising tool for full body scanning with improvements in registration and alignment techniques [25]. Most of the earlier demonstrations have been performed on rotating objects where the Kinect is stationary. However, this may not be possible for large paleontological specimens that are housed in enclosures that cannot be modified.

In this report, we present a method for 3D scanning that is well suited for paleontology and has the following advantages; a) It has an short acquisition time of 60-120s even for large specimens, b) Since the scanner is compact so it can be moved around the specimen on a tripod or adapted to a body-mounted wearable geometry; c) The entire set-up being low-cost and the availability of free scanning and post-processing software.

## Methods

### 3D scanner

A Microsoft Kinect v2 module was utilized as the 3D scanner. It is a module typically used for gesture recognition in gaming and can be modified to capture raw 3D scan data. The Kinect has a 1080p camera operating at 30 Hz that can also capture a regular 2D image that can be used to overlay the color image on the 3D reconstruction. The depth sensor operates at 30 Hz and has a 512x424 sensor. The minimum and maximum depth distances are 0.5 m and 4.5 m, respectively. The horizontal field of view is 70 degrees and the vertical field of view is 60 degrees. These features make the Kinect ideal for scanning large specimens.

### Hardware configuration

A computer or laptop with good graphics processing capabilities (RAM > 4GB, dual-core or multicore CPU) is necessary to handle the 3D data acquisition and rendering. In the current work an ASUS ROG laptop was used with an Nvidia GTX 960M graphics card with 2GB RAM. The system uses a USB 3.0 communication port for high speed data transfer. A frame rate of 16–20 fps was achieved which was ideal for real time capture and registration of the 3D data. Any equivalent of an Nvidia GTX 560M or above will also work although, the frame rate could be slower (<20 fps) on graphics cards with lower processing power.

### Software

Windows 10 operating system was used for better performance with the Kinect sensor and the availability of 3D scanning software like Microsoft 3D Scan. Kinect software development kit (SDK) was also used for capturing raw 3D data. The 3D builder application was used to create the mesh and MeshLab was used to clean, repair, smoothen the mesh. Data was stored in.ply format because of its capability to could capture the actual color of the scene along with the 3D reconstruction. Cleaning of the mesh was carried out by elimination zero area vertices and faces. Repairing the mesh was performed by eliminating non-manifold edges and closing the holes. Finally smoothing was carried out using a Taubin smoothing technique [26] with scaling factors *λ* = 0.8, *μ* = −0.3 and 10 smoothing steps. Additional color smoothing and equalization was also done to remove specimens introduced during the scanning process. Finally, Geomagic Wrap was used for the rendering process.

### Experimental considerations

**Fig 1:**
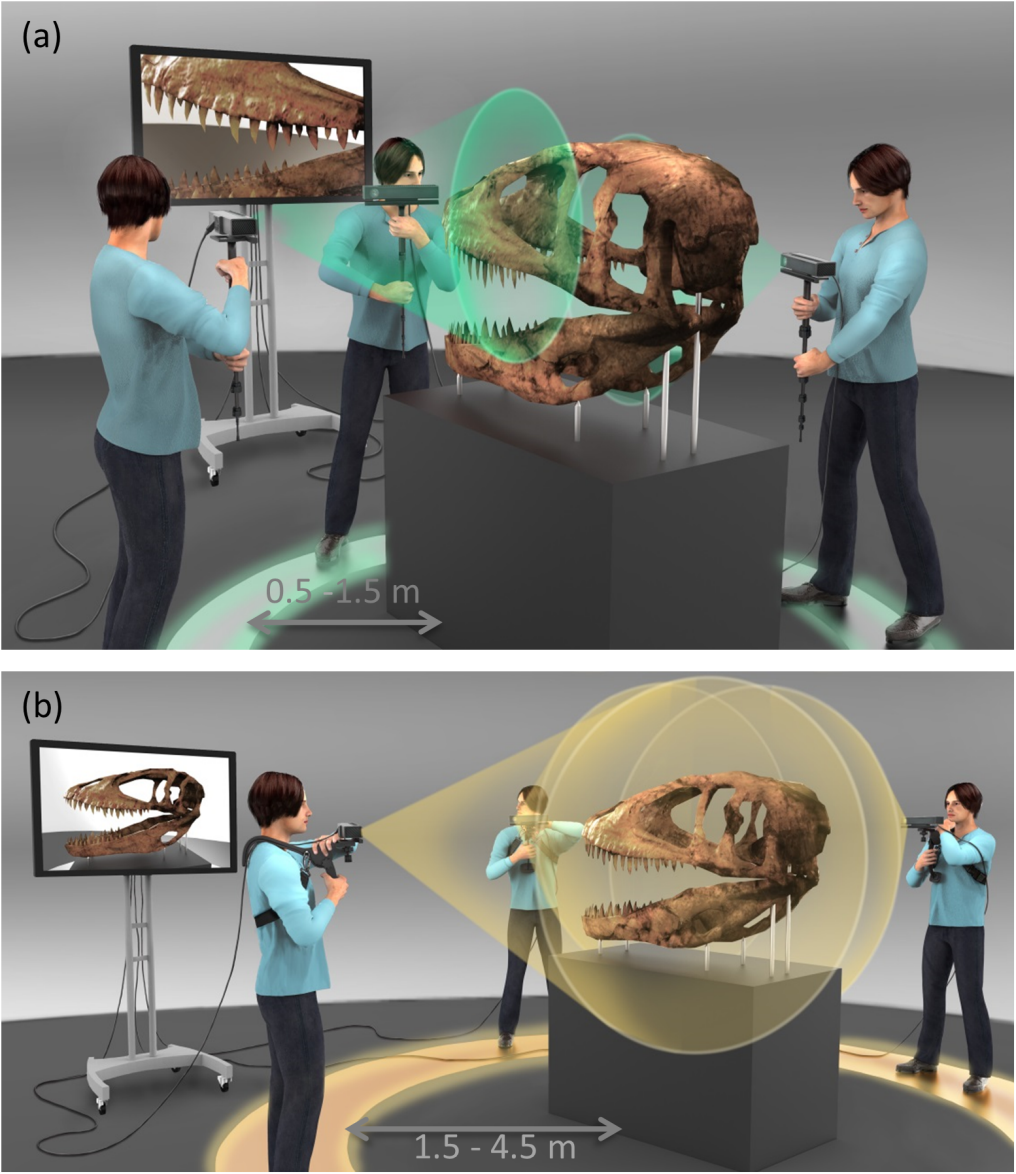
Scanning technique. (a) Small section, high resolution scanning. The user holds a monopod mounted Kinect at close range (0.5–1.5 m) from the target. (b) Large section or complete 360° scanning. The user mounts Kinect on a body supported rig and walks around the artifact (1.5–4.5 m from target) to complete the scan. Sketch by Francis Goeltner.

The scanning was carried out using two methods: Handheld and body mounted. The Kinect was operated in the handheld mode in three different ways. It was mounted on a monopod (Fig 1(A)), held at a suitable height and fixed distance from the object (0.5–1.5 m). In another configuration the user directly held the monopod and performed the scan. In certain cases, a camera dolly was used to minimize vibrations as the Kinect was moved around the object.

The second method involved using a body mounted camera using a shoulder support rig and the user walking around the target (at 1.5–4.5 m) looking at a large screen where real-time registration was carried out (Fig 1(B)). A wireless rolling ball mouse was used to control the software from a distance. This step was important for better mobility of the Kinect sensor and avoided the scenario where the laptop or computer needed to be moved. Appropriate cable lengths also ensured that the Kinect sensor could be easily maneuvered providing clean reconstructions.

### Calibration

Depth calibration was carried out using targets of known thickness at a distance of about 0.5 m. Ultrasound calibration targets with thicknesses from 0.6 mm to 2.5 mm were used to ascertain the minimum resolvable depth at the highest scanning resolution of the Kinect. As can be seen from S1 Fig, 0.6 mm is easily resolvable using Kinect in the best configuration. A target with a thickness of 0.3 mm was not resolved from the background; hence the minimum resolvable depth should be in between 0.3 mm to 0.6 mm.

### 3D printing

The meshes were converted to.stl format in Meshlab and printed by Shapeways, a commercial 3D printing service. Modifications were made to the wall thickness to comply with material and printer parameters. The models were also scaled down by a factor of 8 with respect to the original due to the limitations of the printer. The material used to print the models was PA 2200, a high end plastic used in additive manufacturing.

### Details of the specimen

The specimen studied in the current report is FMNH PR 2081, a *Tyrannosaurus rex* popularly known as SUE at the Field Museum of Natural History in Chicago, IL. SUE is the most complete and best preserved T.rex specimen. The dimensions of the skull were in the range of 1.4 m x 1.3 m and 1 m. Permission to scan was obtained from William Simpson, Head of Geological Collections at the Gantz Family Collections Center and McCarter Collections Manager, Fossil Vertebrates.

## Results

TOF imaging works on the principle that light travels at a finite speed and distance from a target can be calculated by knowing the transit time from the source to the target. In practice the distance can be calculated by time or frequency domain methods. Kinect v2 works on the frequency domain approach which involves the use of modulated light to calculate the phase shift between the emitted and received light to arrive at distance. The following expression can be used to arrive at the depth,

$$d=\frac{\Delta \varphi }{4\pi f}\;c$$ (1)

where, *∆φ* is the phase shift, *d* is the distance between the source and target, *f* is the modulation frequency and *c* is the speed of light. Since the speed of light and the modulation frequency are constants, *d* can be directly obtained from Eq 1 without the need for a post-production scaling process.

This process is carried out for all the pixels on the depth sensor, which in the current case is 512x424 pixels. Subsequent to the data acquisition, the point cloud generation process is performed which essentially consists of a collection of points that describe the surface of the object that is scanned. The point cloud forms the basis for any subsequent conversion into a solid or a mesh that can be used for 3D printing. The sensor is internally calibrated because the lateral dimensions can be known from the optical characteristics of the camera. The axial dimension or depth is derived from Eq 1. A typical mesh can have about 200,000–2,500,000 faces depending upon the volume of the object scanned and the scanning resolution. Subsequent to the generation of the point cloud additional cleaning, repair and smoothing of the mesh was performed in MeshLab as mentioned in the Methods section. The scanning process can introduce holes in the mesh as a result of shadows or lack of reflection from the target due to geometric complexities.

Data was captured using techniques mentioned in the Methods and as shown in Fig 1. For small section scans a monopod mounted Kinect approach was followed whereas for large scans, the Kinect was mounted on the user for the scanning as shown in Fig 1. The specimen FMNH PR 2081 was scanned along with some resin models made from casts of the skull. The skull was removed from the glass enclosure to perform the scanning. The Kinect sensor operates in several modes that require a compromise on the volume of the scan and the depth resolution of the scan. Internal stabilization with handheld option helps in better registration and alignment.

### Small volume, high resolution scan

For volumes in the range of 0.001–0.05 m^3^ the highest resolution mode works satisfactorily. This mode was used to probe smaller portions of the artifact in great detail as shown in 1.

#### Section of the jaw

**Fig 2:**
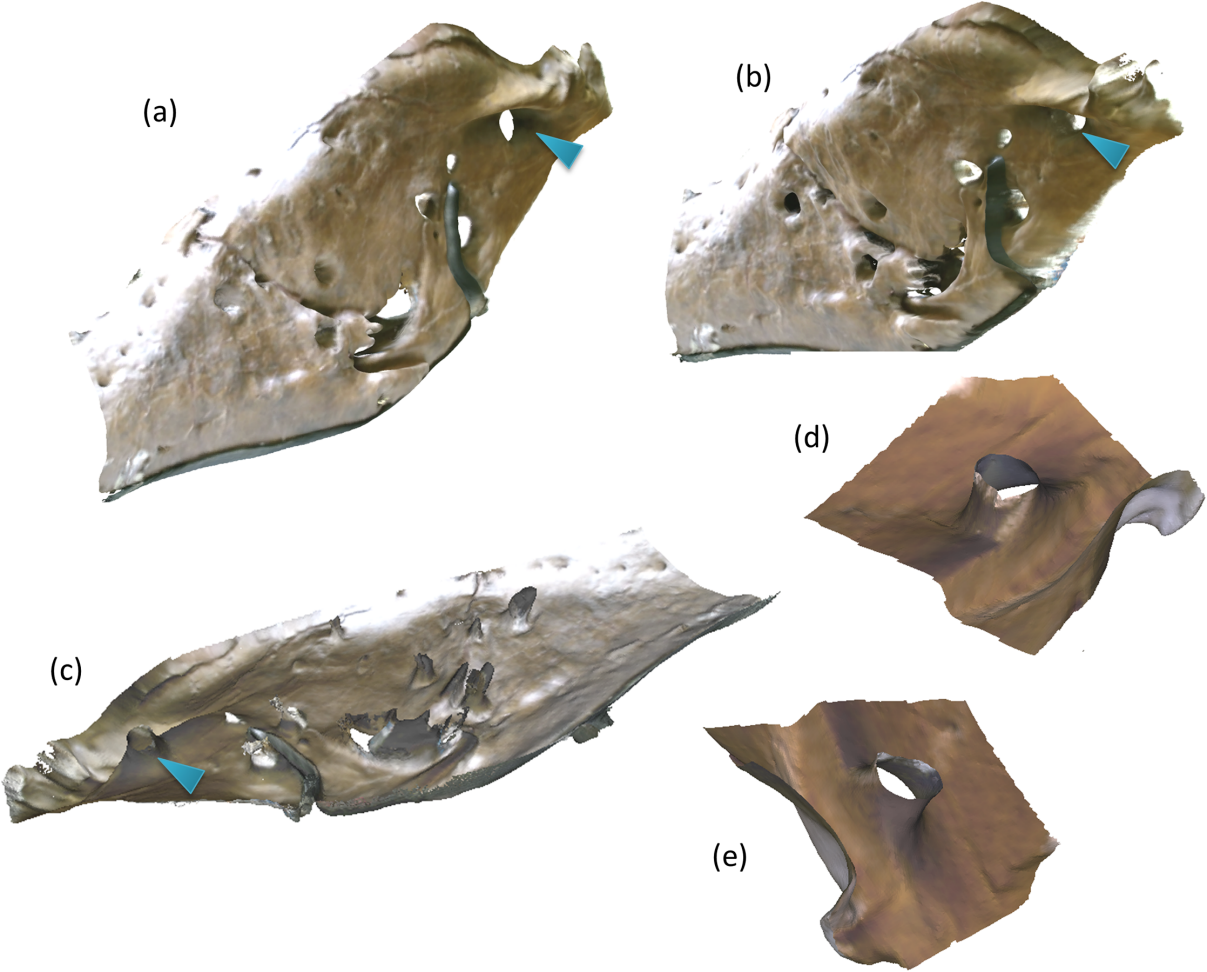
Small volume high resolution scans: Section of the left side of the mandible of FMNH PR 2081. (a) Front view, length of the segment here is about 0.9 m. (b) Side view of the left side of the mandible. (c) Lingual view of the left side of the mandible. (d) & (e) Surangular fenestra which is 2.9 cm deep and about 3.1 cm wide. Number of faces in the mesh in 2(a-c) are 284,273.

The jaw was scanned in the handheld mode moving the Kinect in an arc with a radius of about 0.5–1.5 m from the target in order to get several angles of the section. Fig 2A–2C shows a section of the scan captured in this mode. The left side of the mandible has been captured in detail and the lingual (tongue side) surface structure of the jaw is visible in Fig 2(C). Isolating a single hole, the surangular fenestra in this case, as indicated by the arrow reveals its internal contours as shown in Fig 2D and 2E. The surangular fenestra is about 3.1 cm wide and 2.9 cm deep and quite cylindrical in contrast to other holes which are more angled. Hence, some surface contours can be captured on complex anatomy. It is to be noted the most of the "holes" in the mandible as shown in Fig 2A–2C are not an a regular anatomical feature but are hypothesized to have been caused by bites from other dinosaurs [27] or infections [28].

#### Complete tooth

**Fig 3:**
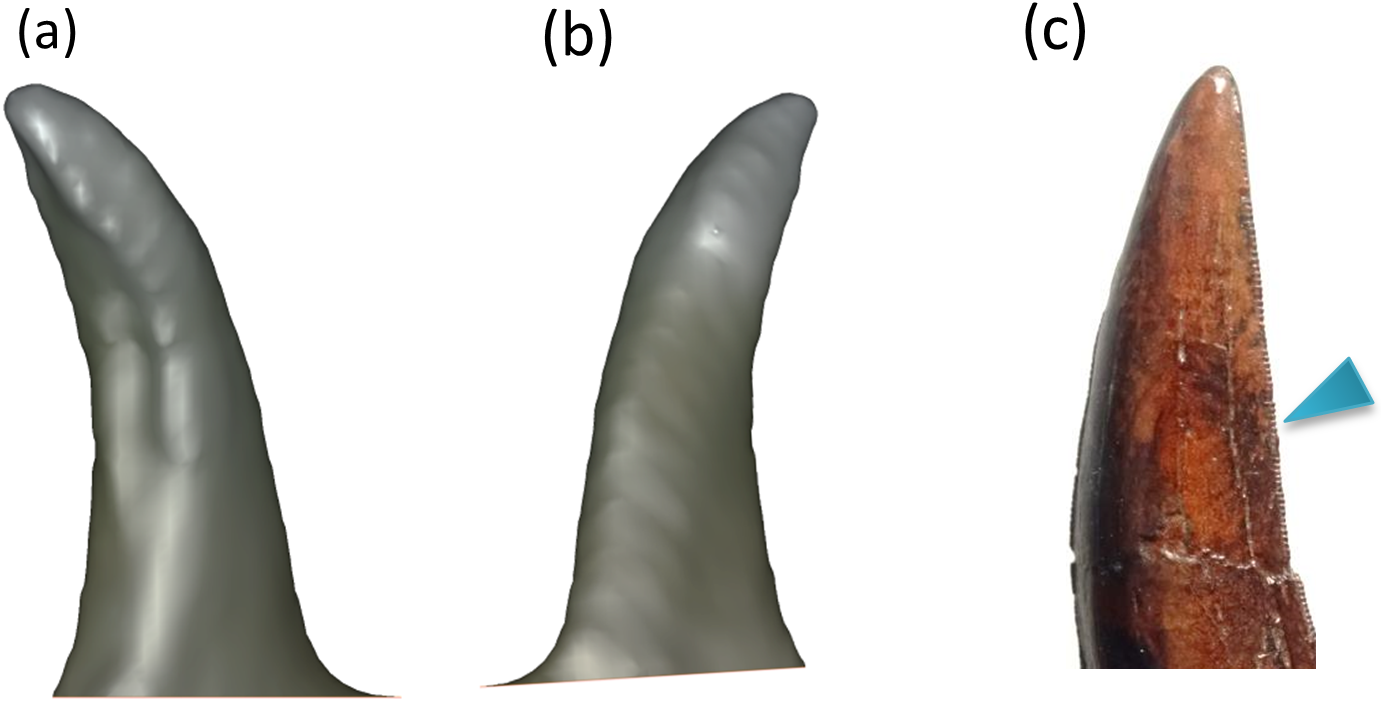
(a-b) Different views of a single tooth. Length of the tooth is about 10.8 cm and the number of faces in the mesh is 6,886. (c) A photograph of a tooth replica showing the serrations.

Tooth morphology plays an important role in studying prey behavior and bite patterns [29] and 3D models can provide significant structural details for analysis. Single teeth were scanned in the small section high resolution mode as shown in Fig 3. The device was operated in the handheld mode with the tooth sample mounted in a sand box placed on a rotating tray for simplified maneuvering. As can be seen from the various angles in Fig 3A and 3B and S2 Fig, the teeth had a complex 3D structure that was beneficial in tearing and cutting their prey [30, 31]. There are several serrations on the edges of teeth, as shown in Fig 3(C), that were not captured due to the depth resolution limitation of the Kinect as they shown to be about 100 microns deep.

#### Other fine features

**Fig 4:**
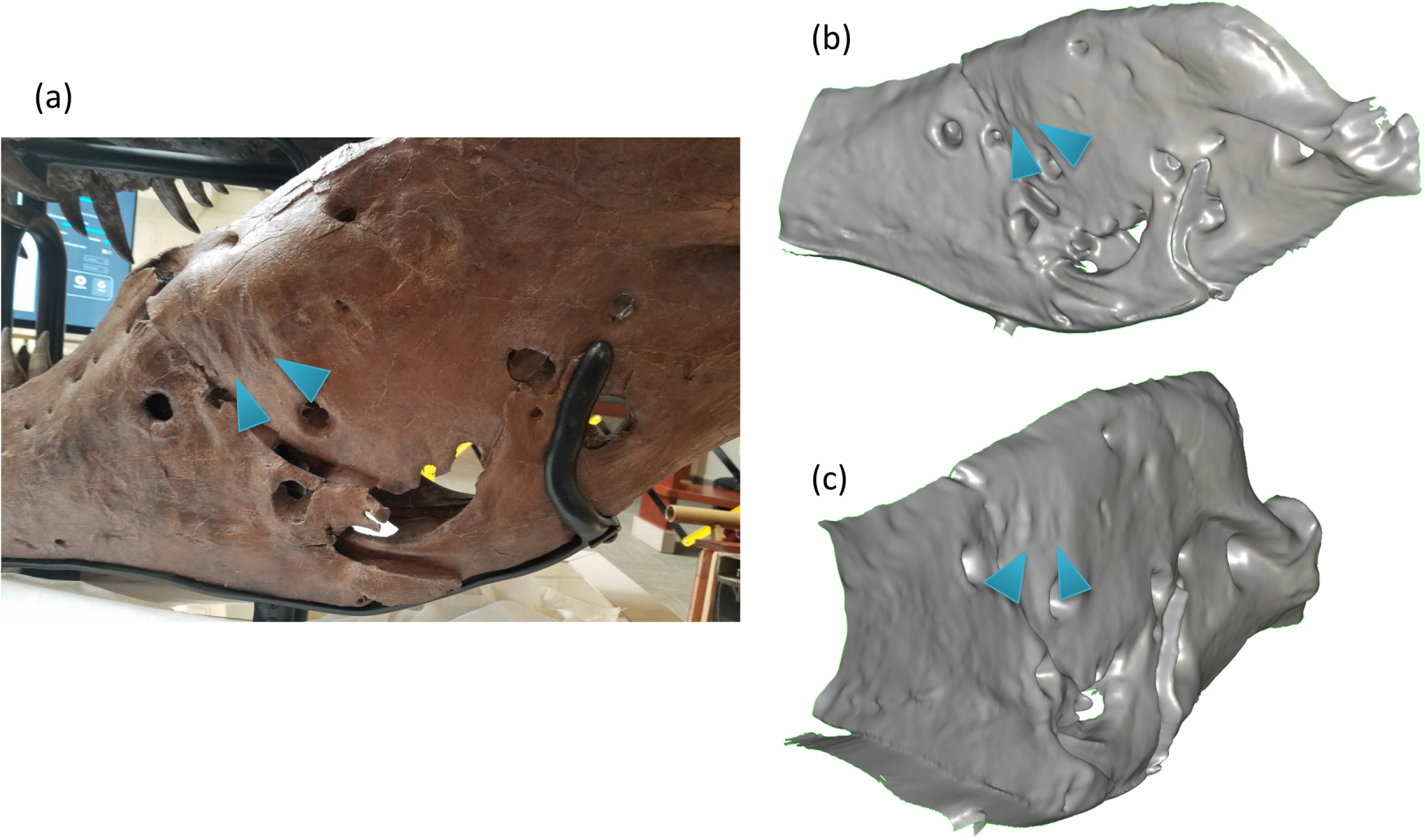
(a) Photograph of the left side of the mandible. (b) & (c) Different 3D views of the mandible. Note the small depressions indicated by markers, which were captured by the depth sensor which were 8 mm in depth.

The device was also able to capture several fine features on the left side of the mandible as shown in Fig 4A–4C. There are indentations on the left side of the mandible that are about 8 mm deep whose source is unknown. Fig 4B and 4C shows different views of the 3D reconstructions of the section and the indentations can be visualized and quantified.

### Large volume, medium resolution scan

#### Entire left section of the mandible

**Fig 5:**
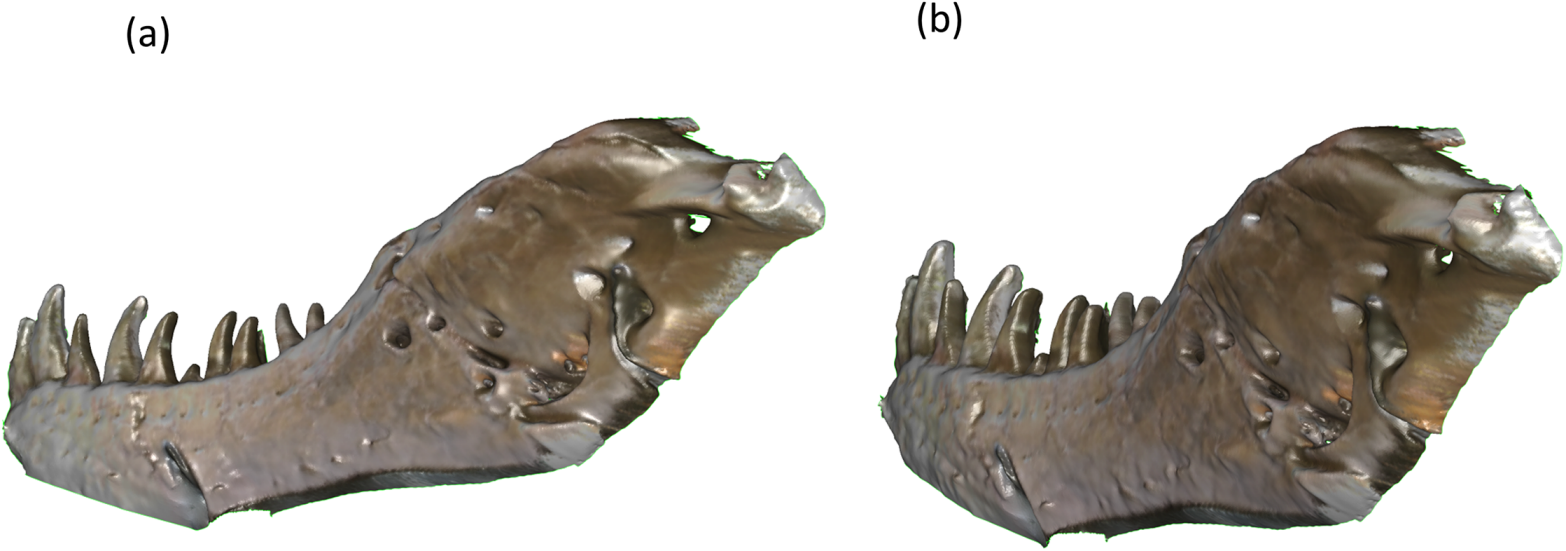
(a) & (b) Different views of the large volume scan of the left side of the mandible. Number of faces in the mesh is 307,343.

Scans were also performed on larger sections of the skull focusing on the entire left side of the mandible as shown in Fig 5. The length of the mandible is about 1.3 m and hence the scanner was translated parallel to the mandible to capture the entire left section. The best scans were obtained when the resolution was set to a mid-range. The volumes scanned in this range were above 0.05 m^3^ up to 1 m^3^. Due to the lower resolution of the point cloud the mesh size in this mode is not significantly different from the scan in Fig 2.

### 360° degree full volume scans

**Fig 6:**
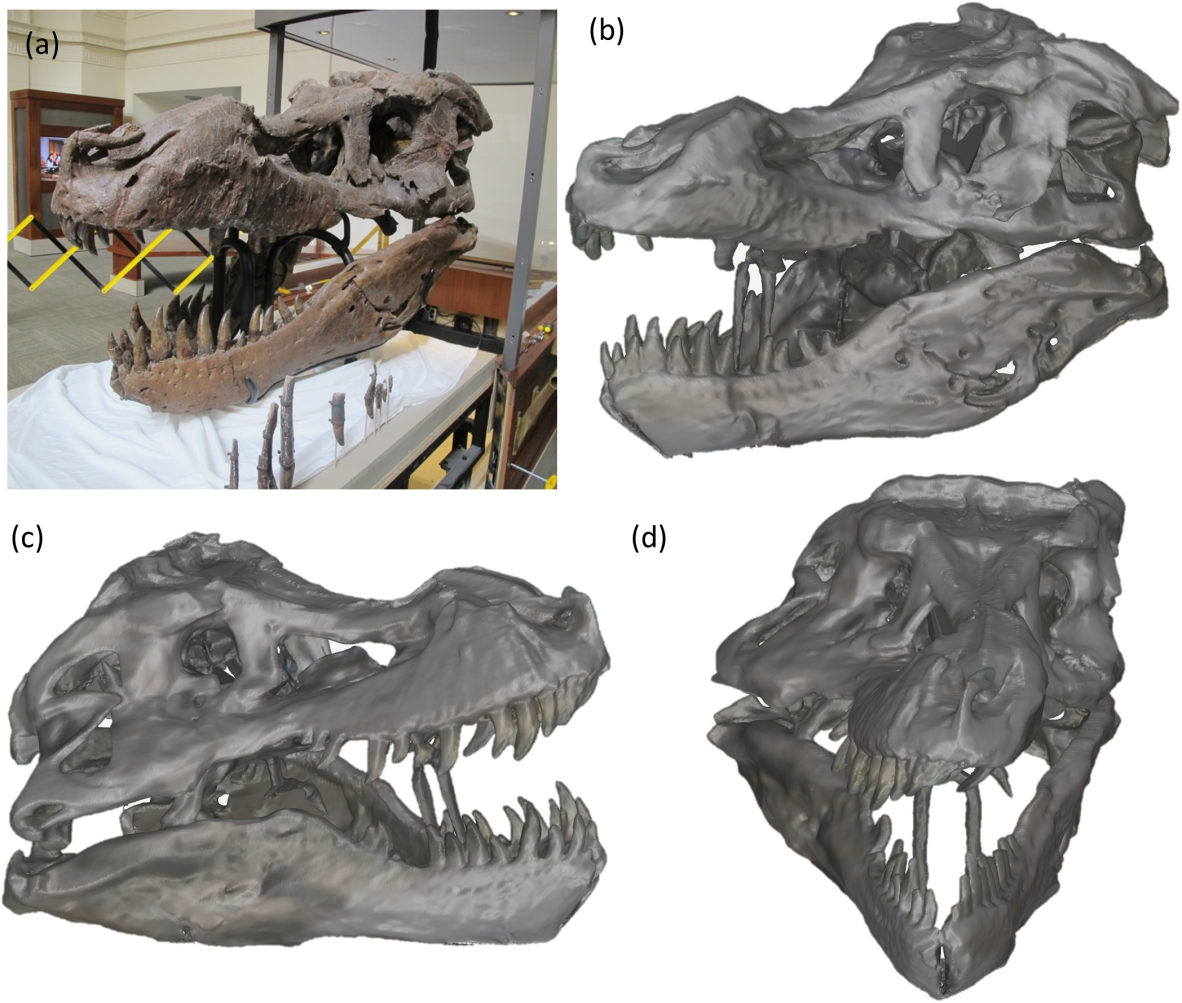
(a) Photograph of skull FMNH PR2081. (b-d) Different views of the 360° scan of the skull in (a). The length of skull is about 1.4 m, the height about 1.3 m and the width at the back of skull about 1 m. Number of meshes in this reconstruction is 1,214,028.

These scans involved complete scanning of the skull taking all angles and registering them real time using the methodology shown in Fig 1(B). The volume of these scans was greater than 1.8 m^3^ which was the entire volume of the skull of FMNH PR 2081 as shown in Fig 6. The entire scan duration was about 120 s. As can be seen from the different views of the 3D reconstructions in Fig 6B–6D) reasonable structural details have been captured in relation to the corresponding 2D image Fig 6(A). A 3D interactable model of the complete skull can be accessed in S3 Fig.

### Validation of scan quality by 3D printing

**Fig 7:**
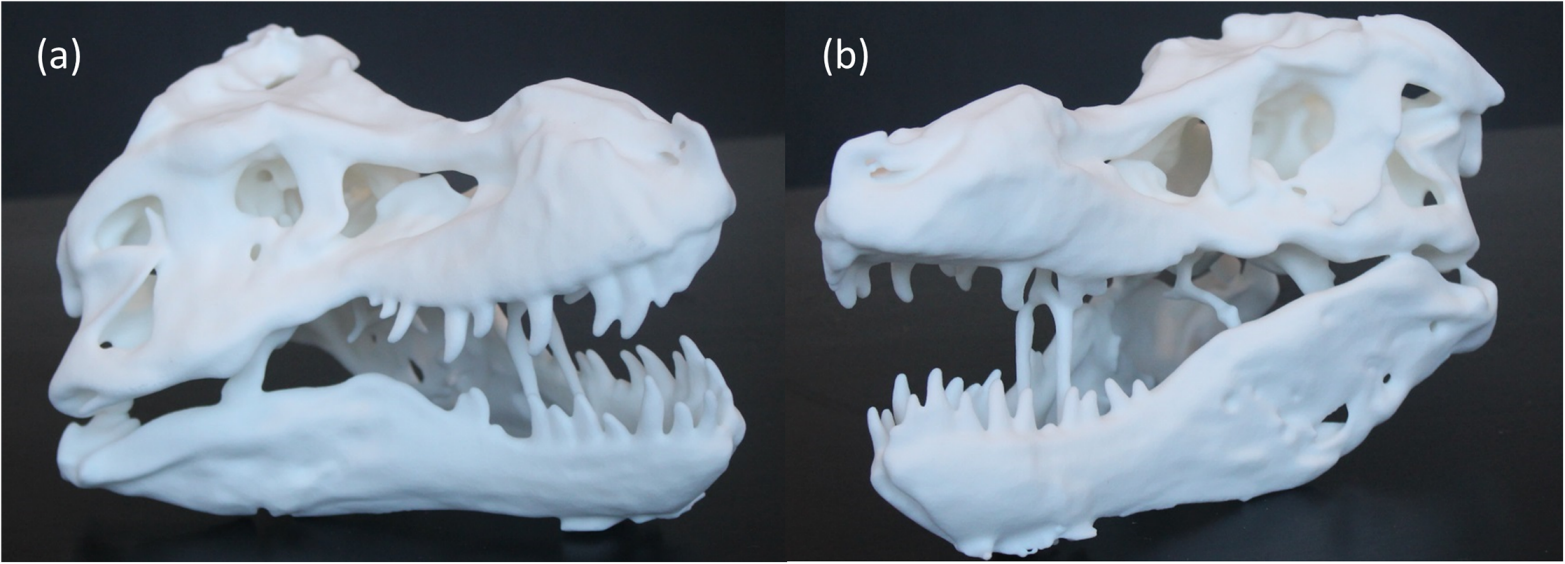
(a,b) 3D printed replicas of the scan shown in Fig 6. The replica is scaled down by a factor of 8 times from the original. The length of the skull replica is about 17.6 cm as compared to the original which is about 1.4 m.

In order to validate the efficacy of the scanning technique, 3D printing of the model in Fig 6 was carried out (Fig 7). Several anatomical measurements were made on the skull and compared to the earlier report by Brochu [32] and a good correlation was observed between the models and the 3D printed replica as can be inferred from Table 1. From these measurements we observe that the 3D models acquired using Kinect had an error under 4.65% when compared to measurements in the report by Brochu. For the 3D printed models the error was in the range of 0.21–4.33% indicating an accurate reconstruction. Except in the case of the width between infratemporal fenestra which have a maximum error of 4.65% the other measurements of the mandibular ramus and the snout- quadratojugal measurements are within 2.57% error. This not only validates the accuracy of the technique but also points to the fact that the model obtained is the original size of the object. In some cases we observe a better correlation of the 3D printed specimen with the measurements in Brochu [32] which is counter-intuitive and could attributed to the small warping or manual polishing artefacts caused during the 3D printing process.

**Table 1:** Comparison of anatomical measurements of FMNH PR2081 using the proposed technique and 3D printing.

Measurement region of the skull	Ref. [32] (m)	Kinect 3D Scan (m)	Percentage error (%)	3D printed specimen (m) (x8 times)	Percentage error (%)
Left mandibular ramus	1.395	1.381	1.00	1.376	1.36
Right mandibular ramus	1.437	1.400	2.57	1.440	0.21
Tip of snout to left quadratojugal	1.407	1.410	0.21	1.392	1.06
Tip of snout to right quadratojugal	1.380	1.380	0.00	1.392	0.87
Width between top of orbits	0.535	0.520	2.80	0.523	2.24
Width between infratemporal fenestra	0.945	0.901	4.65	0.904	4.33

## Discussion

The proposed technique opens up opportunities for research and exploration since it is inexpensive, portable and most importantly, accurate. The Kinect sensor can be procured for about $100 and an adapter for connection to a laptop costs around $ 50. The laptop required costs about $1,500. However, some facilities will already have the hardware that can be used, and if not, once purchased, can then be used for other functions. Hence, this cost is lower than structured light and photogrammetry 3D scanners which can cost from $600 all way up to $50,000 excluding the cost of computers or software. Generally, these techniques are more cumbersome to setup and work well in controlled settings. 3D imaging can help with better visualization of the complex surface structure that can be used for better documentation and educational purposes. The current method should also help in replication of large specimens due to faster scanning time and simple reconstruction procedures. Replicas of exhibits are expensive due to the complex nature of the geometry and this technique should help bring down these costs so that they are available of research and educational use. Although there have been earlier reports of 3D printing replicas of specimens, they have been demonstrated in specimens with much smaller dimensions [33]. Furthermore, with 3D printing becoming more accessible, replicas can be easily produced with short turnaround times. Commercial 3D printing services provide a good platform to print the geometry along with the color variations on the original artifact on a variety of materials from plastics to stainless steel and ceramics.

This technique will help in capturing complex surface structure of specimens that are impossible to capture using regular 2D imaging methods. There have been conflicting reports on the presence of holes in the mandible and jaw of several dinosaur fossils. Some reports claim that they are bites [27] whereas others suggest evidence of infections causing bone damage [28]. A simple 2D image analysis may be inadequate and one could gather rich information from the contour and angulation of natural holes (fenestra) and defects due to trauma or pathology, to come up with a plausible explanation of their origin.

Some drawbacks of the technique can include lower resolution as compared to structured light scanners and the need for rich features when performing 360° complete scans. The lower resolution (about 0.6 mm) is a limit of the modulation frequency which is in turn limited due to electronic switching circuitry. This limitation does not exist in structured light scanning where 50–100 micron depth resolution is possible. As sensors are becoming better, we can envision improved sensors in the near future. The Kinect v2 used in the study outperforms the Kinect v1 in tracking and depth resolution and the trend is expected to continue. However, an improvement in the sensor size and resolution of the Kinect that yields a resolution of about 50 microns will come at an increased cost as compared to photogrammetric methods. The Kinect requires rich set of features to track and register a scene during scanning. For objects that are planar, the 360° scanning does not work very well. However, large paleontological structures can be expected to have a good relative depth and the technique should work more often than not. The current method also relies on the use of a laptop/desktop with larger processing power as compared to photogrammetric methods. This can increase the overall cost of the equipment even though the Kinect itself is relatively cheaper than a standard DLSR camera with medium priced lenses. Hence, the method needs to be chosen for studies that don’t have a stringent resolution requirement but are focused on rapid scanning in large specimens. In the future we should expect to see an integration of the current scanning method to create virtual models in paleontological research that are compatible with the recent trends augmented/virtual reality.

## Supporting information

S1 Fig Calibration test for depth resolution of Kinect. Three test targets whose depth was known were chosen for the study. The Kinect was able to resolve 0.6 mm from the background.
(PDF)

S2 Fig Interactable 3D file of a single tooth. Small volume scan of a single tooth.
(PDF)

S3 Fig Interactable 3D file of entire kull. Large volume scan of the entire skull.
(PDF)
