# Supplementary material for: A method for rapid 3D scanning and replication of large paleontological specimens
Source: PLoS One. 2017 Jul 5;12(7):e0179264. doi: 10.1371/journal.pone.0179264 (PMC5497938; doi:10.1371/journal.pone.0179264)
Supplement: S1 Fig — Three test targets whose depth was known were chosen for the study. The Kinect was able to resolve 0.6 mm from the background. (PDF) [file pone.0179264.s001.pdf]

A method for rapid 3D scanning of large paleontological artifacts

Anshuman J. Das, Denise C. Murmann, Kenneth Cohrn, and Ramesh Raskar

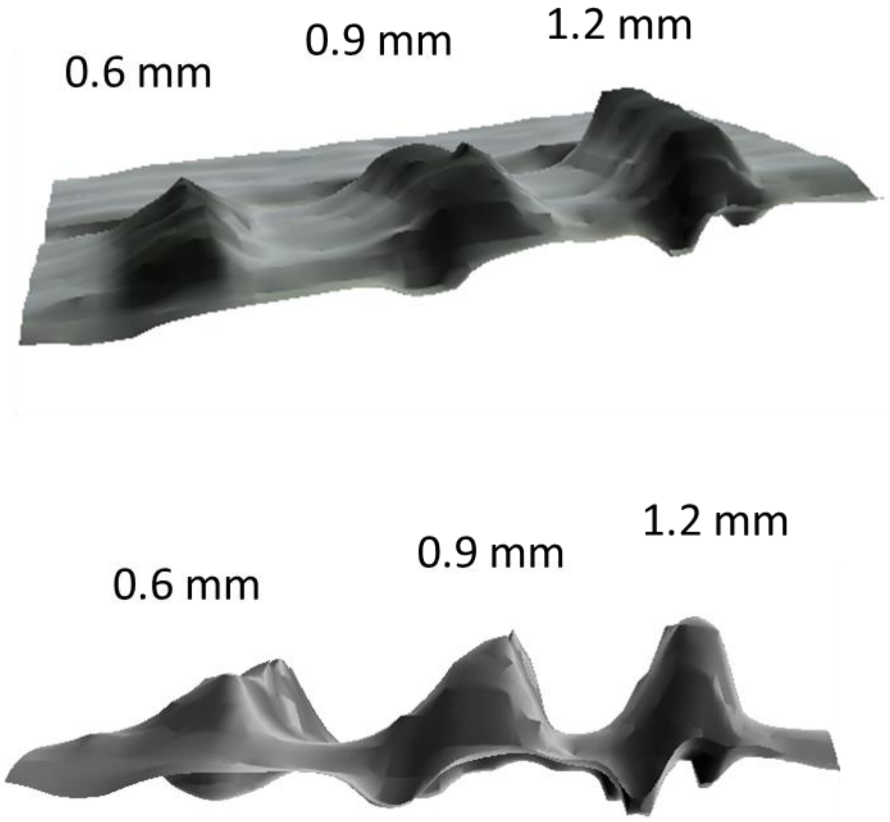

Supplementary Figure 1: Calibration test for depth resolution of Kinect. Three test targets whose depth was known were chosen for the study. The Kinect was able to resolve 0.6 mm from the background.
