# Supplementary figures and images for: A method for rapid 3D scanning and replication of large paleontological specimens
Source: PLoS One. 2017 Jul 5;12(7):e0179264. doi: 10.1371/journal.pone.0179264 (PMC5497938; doi:10.1371/journal.pone.0179264)

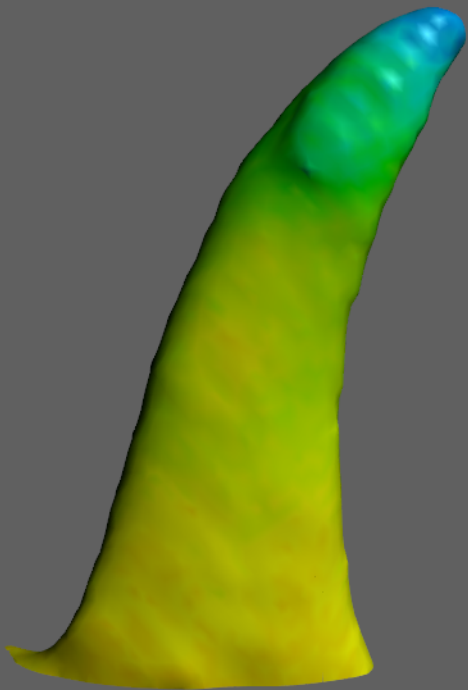

Supplement: S2 Fig — Small volume scan of a single tooth. (PDF) [file pone.0179264.s002.pdf]

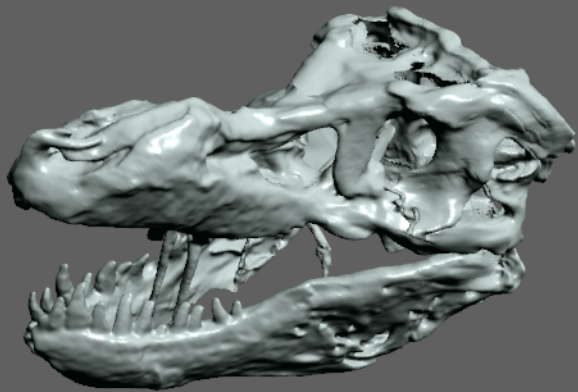

Supplement: S3 Fig — Large volume scan of the entire skull. (PDF) [file pone.0179264.s003.pdf]
